# Systematic review and meta-analysis of remotely delivered interventions using self-monitoring or tailored feedback to change dietary behavior

**DOI:** 10.1093/ajcn/nqx048

**Published:** 2018-02-26

**Authors:** Natalie Teasdale, Ahmed Elhussein, Frances Butcher, Carmen Piernas, Gill Cowburn, Jamie Hartmann-Boyce, Rhea Saksena, Peter Scarborough

**Affiliations:** 1 John Radcliffe Hospital, Oxford, United Kingdom; 2 Medical Sciences Divisional Office, University of Oxford, John Radcliffe Hospital, Oxford, United Kingdom; 3 Oxford School of Public Health; 4 Department of Primary Health Care Sciences, University of Oxford, Oxford, United Kingdom; 5 Centre on Population Approaches for Non-Communicable Disease Prevention, Nuffield Department of Population Health, University of Oxford, Oxford, United Kingdom; 6 University College London Medical School, University College London, London, United Kingdom

**Keywords:** systematic review, diet, nutrition, self-monitoring, tailored feedback, remote delivery, multilevel meta-analysis

## Abstract

**Background:**

Self-monitoring (SM) of diet and tailored feedback (TF) have been suggested as tools for changing dietary behavior. New technologies allow users to monitor behavior remotely, potentially improving reach, adherence, and outcomes.

**Objective:**

We conducted a systematic literature review and meta-analysis to address the following question: are remotely delivered standalone (i.e., no human contact) interventions that use SM or TF effective in changing eating behaviors?

**Design:**

Five databases were searched in October 2016 (updated in September 2017). Only randomized controlled trials published after 1990 were included. Trials could include any adult population with no history of disordered eating which delivered an SM or TF intervention without direct contact and recorded actual dietary consumption as an outcome. Three assessors independently screened the search results. Two reviewers extracted the study characteristics, intervention details, and outcomes, and assessed risk of bias using the Cochrane tool. Results were converted to standardized mean differences and incorporated into a 3-level (individuals and outcomes nested in studies) random effects meta-analysis.

**Results:**

Twenty-six studies containing 21,262 participants were identified. The majority of the studies were judged to be unclear or at high risk of bias. The meta-analysis showed dietary improvement in the intervention group compared to the control group with a standardized mean difference of 0.17 (95% CI: 0.10, 0.24; *P* < 0.0001). The *I*^2^ statistic for the meta-analysis was 0.77, indicating substantial heterogeneity in results. A “one study removed” sensitivity analysis showed that no single study excessively influenced the results.

**Conclusions:**

Standalone interventions containing self-regulatory methods have a small but significant effect on dietary behavior, and integrating these elements could be important in future interventions. However, there was substantial variation in study results that could not be explained by the characteristics we explored, and there were risk-of-bias concerns with the majority of studies.

## INTRODUCTION

A “Western diet” typically consists of intake high in saturated fats, salt, and sugars and low in fruit and vegetables, with most of the population in developed countries not meeting the WHO nutrient recommendations (1). This kind of poor diet is implicated in several chronic noncommunicable diseases (diabetes, some cancers, and cardiovascular disease) (2), and responsible in England for >10% of mortality and morbidity (3); hence, the development of public health initiatives targeting this area (4–6).

The mechanisms by which interventions can induce behavior change have been classified into 93 different techniques in the latest taxonomy iteration (7). One review of components associated with increased effectiveness in dietary and physical activity (PA) interventions found that self-regulatory behavior change techniques (SBCTs), e.g., self-monitoring (SM) and tailored feedback (TF), were associated with positive changes in dietary outcomes and differences in how interventions were delivered (provider, settings, or modality); moreover, study population characteristics did not appear to be associated with differences in effectiveness (8). Additionally, synergistic effects may occur when SBCTs are combined with other methods that target future performance, particularly those derived from control theory (9).

For diet and PA interventions, SM requires the recording of behavior, e.g., intake or activity, by an individual to actively track trends (10), primarily initiated to motivate modification of unwanted dietary or PA behavior. For SM to be successful, consistent and frequent recording is required (11), with self-evaluation being the next step, followed by self-reinforcement (12, 13). To achieve the recommended behavioral change, i.e., to promote meaningful alterations and maintain wanted behaviors, individuals must analyze their actions, change them accordingly, and preferably repeat the cycle of evaluating behavior against their incorporated standards (13).

On the same continuum as SM, providing TF [where a user's unique characteristics are utilized—e.g., previous actions (14)] can be effective in changing future behavior. Compared to generic information, TF has the potential to provide more individualized information that is perceived as more salient by the user, increasing the likelihood of adhering to such advice (15, 16).

We did not distinguish between interventions that use SM or TF in this review, though they differ in who is providing the results of previous behavior, because they often intertwine and hence can be difficult to separate out; indeed, under the behavior change technique classification, they come under the same cluster (7). This is especially pertinent when technology is used, as many apps contain both SM and TF as integral components, e.g., an app that provides users with a breakdown of the nutrient composition of their meal after they enter the foods they have eaten.

### Aim

Our systematic review aimed to answer the question: are remotely delivered interventions that use SM or TF effective in changing eating behaviors?

## METHODS

We used initial searches to identify keywords in PubMed to develop a strategy for adaptation to other databases (Embase, CENTRAL, PSYCHINFO, and Web of Science). The search, which was conducted in October 2016 and updated in September 2017, was restricted to those articles published after 1990 in peer-reviewed literature, in English, French, or Spanish. The protocol was placed in advance on PROSPERO: CRD42016042015 (http://www.crd.york.ac.uk/PROSPERO/display_record.asp?ID=CRD42016042015) and contains the search strategies used.

In order to be included, trials needed to use a remotely delivered (i.e., any standalone method that does not use direct human support) dietary SM or TF intervention in the intervention arm only and contain outcomes pertaining to most dietary consumption behaviors. We limited our study type to randomized controlled trials. Studies were excluded if they were based on populations that included children <18 y of age; those with impairments leading to disordered eating (e.g., anorexia nervosa); mixed behavior interventions (e.g., diet and exercise); interventions delivered face-to-face, in groups, or via telephone or video calls; feedback solely tailored to characteristics other than previous dietary behavior (e.g., feedback tailored by demographics); outcomes only measuring weight, calories, micronutrients (except salt), total carbohydrates, or protein (these outcomes were not included as, depending on study aims and populations of interest, these outcomes could be intended to increase, decrease, or remain the same. However, all other dietary consumption behaviors were included as outcomes for the review).

Three assessors independently screened consequent samples of 5%, calculating interrater agreement with Cohen's and Fleiss’s κ. At 10%, substantial agreement had been reached (Cohen's κ = 0.62–0.87, Fleiss’s κ = 0.67–0.72) (17) and the remainder was split between the assessors. Two reviewers extracted the study characteristics, intervention details, and outcomes and assessed the risk of bias using the Cochrane tool (18). Subgroup analyses were conducted stratified by risk of bias, with studies categorized as being at a high risk of bias if they were rated as a high risk for any bias included in the Cochrane tool with the exception of performance bias (due to the nature of the intervention, virtually all of the studies did not blind the participants to their intervention status).

### Data synthesis

For each study, data were extracted on the first reported measures either during the intervention period or instantly after it to assess the immediate impact of the intervention. For each dietary outcome, we extracted data on the mean and SD within the control and intervention groups. Where results were reported for >1 control or intervention group, the mean and SD were combined using standard methods described in the Cochrane handbook (18). In some cases, data were extracted on the mean and SD in the change of outcome between intervention period and baseline, but only when data on mean and SD of the outcomes themselves were not available; this was not chosen as the primary outcome as it was only adequately reported in a small minority of studies. All results were converted to standardized mean differences (SMDs) using Hedge's *g* statistic, as described in the Cochrane handbook (18). For dietary outcomes where the intervention aims to reduce consumption (e.g., saturated fat intake), we multiplied the SMD by –1, so that in each case a positive SMD represented an improvement in diet.

All of the dietary outcomes were included in a 3-level, random-effects meta-analysis, with both individuals and dietary outcomes nested in studies. This method explicitly accounts for correlation within studies of different dietary outcomes (19). Heterogeneity was measured using the *I*^2^ statistic. The inclusion of multiple outcomes from single studies precludes the use of funnel plots to assess the risk of publication bias, and therefore we conducted a multilevel (outcomes nested in studies) meta-regression of the effect of standard error on effect size; in the absence of publication bias, these 2 variables should not be related. As a sensitivity analysis, we conducted a “one study removed” analysis to assess whether our meta-analysis results were overly influenced by any of the included studies. We explored reasons for heterogeneity by conducting univariate multilevel (outcomes nested in studies) regression analyses of the impact of the following variables on the effect size: risk of bias; type of dietary outcome (fruit and vegetables, fatty acids, or other); geography (Europe, United States, or other); mode of delivery of intervention (mobile phone, website, or other); length of intervention period; population type (general population, or specified by cardiovascular risk factor, e.g., overweight); and method of diet measurement (food diaries, food frequency questionnaires, or other). All analyses were conducted in R version 3.2.2, using the metafor and lme4 packages (20–22).

## RESULTS

**FIGURE 1:**
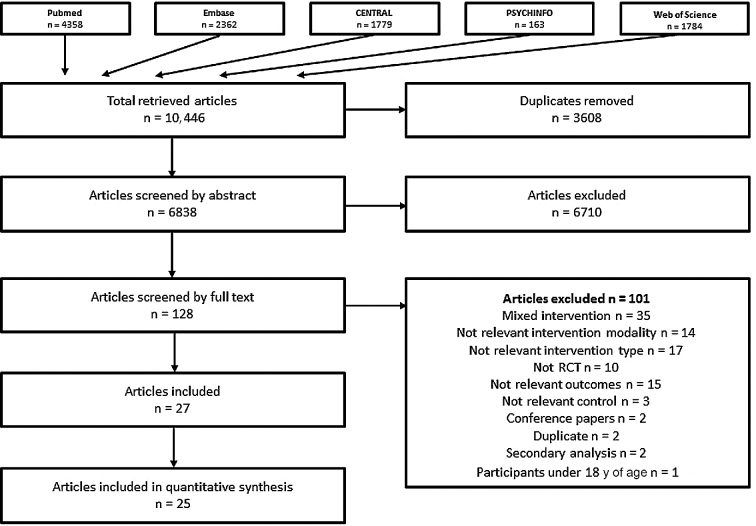
Study selection. RCT, randomized controlled trial.

As shown in **Figure 1**, after duplicates were removed, we retrieved 6838 articles. After abstract and paper screening, this was narrowed down to 27 articles (see **Table 1**) (23–49) reporting on 26 studies containing 37 interventions for inclusion, of which 23 studies (23, 24, 27–39, 41–49) were included in the meta-analysis.

**TABLE 1:** Characteristics of included studies^1^

Study first author, year, country (ref)	Population	Length (mo)	Intervention(s)	Tailored on	Comparison(s)	Outcomes	Measurement method
Alexander, 2010, USA (23)	2513 healthcare members	12	1. “MENU” program (website)	Latest FFQ	General nutritional information website	F + V (serv)	2-item FFQ
			2. Program + e-mail counselling				
Armitage, 2001, UK (24)	801 hospital workers	5	A letter with an additional sentence on current fat intake	Baseline FFQ	A general nutritional information leaflet	Fat %/sat fat (g)	63-item FFQ
Atienza, 2008, USA (25)	36 participants from general population	2	PDA program (notified to SM 2 times/d)	PDA FFQ	General nutritional information material	Vegetables (serv)/fiber (serv)	FFQ, unknown length
Block, 2004, USA (26)	491 low-income women	9	“Little by Little” CD-ROM (once)	CD-ROM survey	Stress-management CD-ROM	F + V (serv)	1-d dietary recall
Brug, 1996, Netherlands (27)	507 oil company employees	1.5	A tailored report	Baseline survey	General nutritional information material	Fat (score)/F + V (serv)	30-item FFQ
Brug, 1998, Netherlands (28)	762 participants from general population	2	1. A tailored report	Baseline survey	General nutritional information material	Fat (score)/F + V (serv)	32-item FFQ
			2. Two tailored reports	Latest survey			
Campbell, 1994, USA (29)	558 patients	4	A tailored report	Baseline survey	1. An untailored report	Fat (g)/sat fat (g)/F + V (serv)	28-item FFQ
					2. No information		
Campbell, 1998, USA (30)	526 low-income women	3	“Sisters at Heart” computer program (once)	Survey in program	No information	Fat (score)	16-item FFQ
Gans, 2009, USA (31)	1841 low-income adults	7	1. A tailored report	Baseline survey	An untailored report	Fat (score)/F + V (serv)	FFQ, unknown length
			2. The tailored report split into 4	Latest survey			
			3. Four retailored reports				
Gans, 2015, USA (32)	2525 employees from 43 worksites	8	1. Three tailored reports	Latest survey	3 untailored reports	Fat %/F + V (cups)	FFQ, unknown length
			2. Three tailored reports + 3 videos				
Heimendinger, 2005, USA (33)	3402 callers to cancer hotline	12	1. A tailored report	Baseline survey	An untailored report	F + V (serv)	1-item FFQ
			2. Four tailored reports	Latest survey			
			3. Four retailored reports				
Huang, 2006, Australia (34)	497 internet shoppers	5	Feedback while shopping online	Food selected	General nutritional information website	Sat fat %	Food purchased
Kerr, 2016, Australia (35)	247 participants from general population	6	1. Two feedback texts (once)	Baseline food diary	Wait listed	F + V (serv)/SSB (serv)/EDNP (serv)	4-d food diary (photos using mobile app)
			2. The 2 initial texts before weekly motivational texts				
Kroeze, 2008, Netherlands (36)	442 participants from general population	6	1. CD-ROM program	Baseline FFQ	General nutritional information material	Fat (g/%)/sat fat (g/%)	35-item FFQ
			2. A tailored report				
Lutz, 1999, USA (37)	710 medical insurance subscribers	6	1. Four tailored reports	Baseline survey	1. Four untailored reports	F + V (serv)	17-item FFQ
			2. The reports also including goalsetting		2. No newsletters		
Mummah, 2016, USA (38)	17 overweight iPhone users	3	“Vegethon” mobile app (requested daily use)	N/A	Wait-listed	Vegetables (serv)	28-item FFQ
Oenema, 2005, Netherlands (39)	782 employees from 7 worksites	0.75	Program (CD-ROM/intranet)	Survey in program	1. General nutritional information material	Fat (score)/F + V (serv.)	49-item FFQ
					2. No information		
Poddar, 2010, USA (40)	294 students	5	Internet course (requested daily use)	Latest FFQ on course	No course	Dairy (serv)	7-d food diary
Raats, 1999, UK (41)	171 university staff	4	A tailored report	Baseline food diary	No report	Carbs %/fat %/protein %	7-d food diary
Springvloet, 2015 a & b, Netherlands (42, 43)	1349 participants from general population	9	1. “Basic” internet program (requested use 3 times/wk)	Past behavior in program	General nutritional information material	Fruit (pieces)/veg (g)/sat fat (points)/high-energy snacks (pieces)	66-item FFQ
			2. “Plus” program				
Tapper, 2014, UK (44)	100 people wanting to improve diet	6	“Health Values ” internet program (requested weekly use)	Latest online FFQ	No information	F + V (cups)/sat fat (g)/added sugar (g)	55-item FFQ
Turnin, 1992, France (45)	105 diabetic subjects	12	“Diabeto” program (Minitel)	Meals entered	Wait-listed	Carbs %/fat %	3-d diet analysis by dietitian
Turnin, 2001, France (46)	557 obese people	12	“Nutri-Expert” program (Minitel)	Meals entered	7 dietitian/doctor visits	Carbs %/fat %/protein %	3-d food diary
Wright, 2011, Australia (47)	178 people with cardiovascular risk factors	3	3 reports	Latest FFQ	1. Two group education sessions by dietician	Sat fat (g)/fiber (g)/F + V—not potatoes (serv)/grain (serv)	1. 63-item FFQ
					2. Wait list		
							2. 7-d food diary
Carfora, 2017, Italy (48)	244 students	0.25	Daily text messages encouraging self-monitoring of red meat consumption	N/A	No contact	Red meat consumption	Food diaries
Celis-Morales, 2017, 7 European countries (49)	1607 adults	6	1. Personalized feedback on diet	Baseline FFQ	General dietary advice	Fruit, vegetables, whole grains, oily fish, red meat, salt, total fat	157-item FFQ
			2. Personalized feedback on diet + phenotype				
			3. Presonalized feedback on diet + phenotype + genotype				

^1^EDNP, energy dense, nutrient poor; FFQ, food-frequency questionnaire; F + V, fruit and vegetables; N/A, not applicable; PDA, personal digital assistant; ref, reference; sat fat, saturated fat; serv, servings; SM, self-monitoring; SSB, sugar-sweetened beverages.

### Population

All studies were performed in high-income countries, with the majority (*n* = 11) held in the United States (23, 25, 26, 29–33, 37, 38, 40), followed by the Netherlands (*n* = 5) (27, 28, 36, 39, 42, 43), with the rest comprised of Australia, the United Kingdom, Italy, France, and a single trial conducted in multiple European countries. There were 21,262 participants in total. Where the information was provided, ages ranged from 18 to 79 y with the average number of participants at baseline being 818 (median 517), dropping to 598 (median 456) at study completion. Participants were found from: the general population in 6 studies (25, 28, 35, 36, 42, 43, 49); those with risk factors such as obesity or diabetics (*n* = 4) (38, 45–47); employees (*n* = 5) (24, 27, 39, 41); recruited from healthcare networks, e.g., primary care patients (*n* = 4) (23, 29, 33, 37); those with low income (*n* = 3) (26, 30, 31); and the 4 remaining used students (40, 48), internet shoppers (34), and those who wished to improve their diet (44).

### Delivery of intervention

The interventions, which had a mean and median length of 6 mo, were carried out in a variety of ways: paper reports, letters, or booklets were used by 11 (24, 27–29, 31–33, 36, 37, 41, 47), 1 using this in conjunction with a computer (36). The internet was used in 7 cases (23, 34, 39, 40, 42–44, 49)—again, 1 study used this method alongside offline computer use (39). Those solely using computers accounted for 2 studies (26, 30), with the remainder using either handheld devices (*n* = 3), divided up into mobile phones (35, 38, 48) and personal digital assistants (25), or Minitel (*n* = 2)—a French version of teletext (45, 46).

The majority of the studies used TF solely; only 2 studies used SM on its own (38, 48). Tailoring was rarely based on observed behavior in the intervention, happening in 4 occurrences (34, 42, 43, 45, 46), but on data collected through surveys (*n* = 18) (23–33, 36, 37, 39, 40, 44, 47, 49) or twice with food diaries (35, 41). The control in 16 cases was given general untailored nutritional information (23–25, 27–29, 31–34, 36, 37, 39, 42, 43, 48, 49), 3 of which also had another control group receiving no information (29, 37, 39). No information was provided to the control group in 4 studies (30, 40, 41, 44). In 4 studies the intervention was provided to the control group at the end of the study (35, 38, 45, 47); additionally, in 1, another part was given group education instead (47). The remaining 2 were given either health professional visits (46) or nonnutritional information, i.e., stress management (26).

### Outcomes

Multiple dietary outcomes were collected in most of the studies included in the meta-analysis, including: total fat (*n* = 11) (24, 27–32, 36, 41, 45, 46), saturated fat (*n* = 8) (24, 29, 34, 36, 39, 42–44, 47), fruit (*n* = 8) (27, 28, 32, 35, 39, 42, 43, 47, 49), vegetables (*n* = 9) (27, 28, 32, 35, 38, 39, 42, 43, 47, 49), fruit and vegetables together (*n* = 7) (23, 29, 31, 33, 37, 44, 49), and assorted others (*n* = 7) (35, 42–44, 47–49). No studies included fiber as an outcome. These dietary outcomes were measured in 2 main ways: either food-frequency questionnaires (*n* = 17) (23–25, 27–33, 36–39, 42–44, 49) or via a food diary (*n* = 7) (26, 35, 40, 41, 45, 46, 48). In the remaining 2 studies, 1 used both the questionnaire and the diary (47), whereas purchased food was assessed in the other (34).

Three trials were excluded from the meta-analysis as we could not extract numeric outcome data (25, 26, 40); 2 of these showed no change in the intake amount of either fruit and vegetables (26) or dairy (40). Atienza showed an increase of vegetables by 1.5–2.5 servings/d (*P* = 0.02) and in fiber by 3.7–4.5 servings/d (*P* = 0.10) (25).

### Bias

**FIGURE 2:**
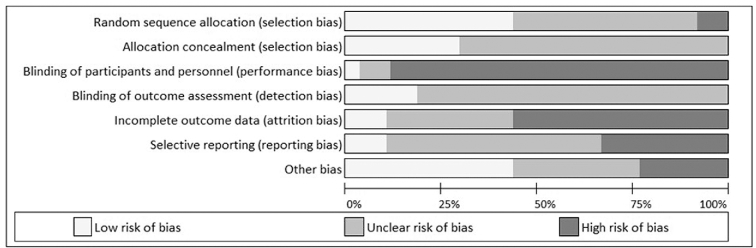
Bias assessment using the Cochrane assessment of bias tool.

As shown in **Figure 2**, most studies were found to have an unclear risk of bias in the domains of random sequence generation, allocation concealment, blinding of outcome assessment, and selective reporting due to a lack of clarity about how randomization, blinding, or analysis was undertaken. No studies had a high risk of bias in either allocation concealment or blinding of outcome assessment. Due to the nature of the interventions, it is difficult to blind participants, and hence most studies were found to have a high risk of bias in the related section. If this domain is disregarded, only 5 studies did not contain high risk of bias judgments.

### Meta-analysis

**FIGURE 3:**
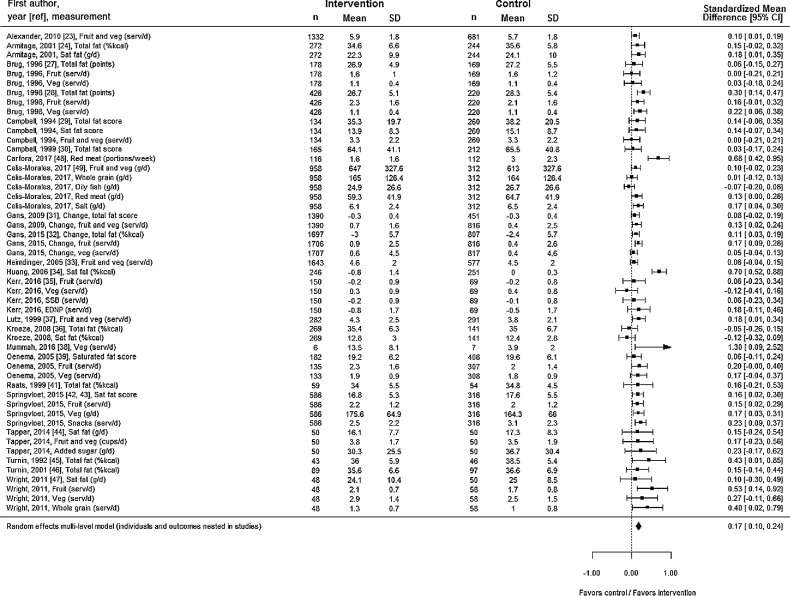
Forest plot of the 51 dietary outcomes nested in 23 studies included in the meta-analysis. Fat scores or points are as described in Brug et al., 1998 (28): “The fat score that ranges between 12 and 60 is the result of a short [FFQ] in which the frequency of use and portion size of the 12 main fat sources in the Dutch diet are assessed”; Campbell et al., 1994 (29): “Dietary fat and saturated fat scores were obtained by multiplying frequency of consumption (calculated as servings per day) by portion data for each item and summing the items”; Campbell et al., 1999 (30): “Dietary fat scores were obtained by multiplying frequency of consumption adjusted to daily intake (3, 2, 1, 0.5, 0.14, 0.07 and 0) by fat content per serving of each item and summing items”; Gans et al., 2009 (31): “The FHQ fat summary score was calculated by taking the mean of all behavioral FHQ questions … response categories for the behavioral questions were: 0 = almost always, 1 = often, 2 = sometimes, 3 = rarely, and 4 = never”; Oenema et al., 2005 (39): “Answers to the [FFQ] items were converted into a fat score ranging from 0 to 80, reflecting total saturated fat intake”; Springvloet et al., 2015 (42, 43): “Saturated fat intake was measured with [an FFQ] … Based on this questionnaire, fat points were calculated … The total ‘fat score’ was based on 35 … food products [to which] … fat points were assigned for each product group, ranging from zero … –5 (…summed up to create a total fat points measure).” EDNP, energy dense, nutrient poor; FFQ, food-frequency questionnaire; FHQ, food habits questionnaire; Sat, saturated; serv, servings; SSB, sugar-sweetened beverage; Veg, vegetables; %kcal, percentage of total.

The multilevel meta-analysis revealed a pooled SMD of 0.17 (0.10, 0.24; *P* < 0.0001), indicating a significant improvement in diets as a result of tailored feedback and/or self-monitoring of diets (**Figure 3**). An SMD <0.2 is sometimes described as a small effect (50). The *I*^2^ statistic for the meta-analysis was 0.77, indicating that 77% of the variance in the final result was due to between studies variance.

Multilevel regression (outcomes nested in studies) showed that the standard error was strongly positively associated with effect size, indicating potential publication bias. However, 1 study (38) was an outlier in the regression, with very large SE and effect size compared to the other results. Therefore, we conducted the regression analysis again with this study excluded. The results showed no effect of SE on effect size (β = 1.11, SE = 0.73, *P* = 0.131), indicating no evidence of publication bias. The “one study removed” sensitivity analysis revealed that no single study unduly influenced the results of the multilevel meta-analysis. The pooled effect size in the sensitivity analyses ranged from 0.12 to 0.18 and the *P* values were always <0.001. The subgroup analyses stratified by risk of bias showed very little difference in studies with low risk of bias [SMD 0.17 (0.05, 0.29), *P* < 0.01] and high risk of bias [SMD 0.17 (0.08, 0.26), *P* < 0.001] (see **Supplemental Figure 1** and **Supplemental Figure 2**). Our multilevel regression analyses exploring reasons for the heterogeneity in results found only 1 variable where differences in effect size were significant at the *P* < 0.05 level. Studies that measured diet quality using means other than food diaries or food-frequency questionnaires produced larger results (*P* = 0.028). The only other result with borderline significance was that results for dietary outcomes other than fruit, vegetables, or fatty acids tended to be larger (*P* = 0.077) (see **Supplemental Table 1** for full results).

## DISCUSSION

Our review showed a positive but small change in diet as a result of SM or TF (based predominantly on studies of TF), although with high heterogeneity between results. That is to say, remote interventions using self-regulation methods do influence dietary change for the better and, potentially, if this effect was extrapolated over a population, it could produce a significant impact (51). This is despite potential barriers such as cost (52)—following dietary recommendations has been found to cost more and hence can become unaffordable amongst lower socioeconomic classes (53, 54)—as well as restrictions that participants face on time, motivation, social support, organizational demands, and emotional availability (55, 56).

As this review shows that approaches not requiring instantaneous personal contact can positively impact on diet, this has implications for rolling out and integrating digital health interventions into mainstream clinical practice. Media that have a high potential reach and widespread usage, such as supermarket loyalty cards (57) and health apps—both mobile (58) and online (5, 59)—can potentially raise adherence and the measurement accuracy of SBCTs. In addition, they can be programmed to provide advice that accommodates personal needs and preferences, leading to potential improvements in associated health behaviors (56, 59, 60). It is easier to implement digital interventions at scale (8, 56), but most of the evidence identified in this review is from studies that used more traditional research media (e.g. paper diaries).

### Other literature

Although there have been previous systematic reviews that have looked at SM and TF interventions (9, 10, 15, 60–67), our review was more inclusive on population characteristics, outcomes, and delivery methods. In contrast, prior reviews have the following limitations: *1*) they have focused on those without health difficulties (9) or a narrow population such as obese participants (10); *2*) they have concentrated on the intention to change (67) or weight loss (60); *3*) they were restricted to particular food groups (63); *4*) they used only specific delivery media (61, 66); and *5*) they did not discriminate between a wide variety of behavioral techniques (65) or did not isolate the dietary component of the intervention (15, 62). Our work shows similar results to these systematic reviews discussed below which have shown small positive effect sizes on several discrete outcomes (9, 62, 64). Additionally our review extends this, through combining a broad range of dietary outcomes to show an overall effect, by using the technique of multilevel meta-analysis (19), enabling us to cope with within-study correlations between study outcomes.

The review by Broekhuizen et al. (62) looked into computer tailoring of education for nutrition and PA compared to generic or no information. In the dietary domains, a favorable significant effect was found in fat (81%), fruit and vegetables (83%), and both studies on fiber. However, this was not the case in interventions on grain, added sugar, or dairy, which were comprised of one study each.

Eyles and Mhurchu (64) analyzed the long-term effectiveness (≥6 mo) of nutritional TF. For fruit and vegetables, 4 studies comparing TF against generic information showed a weighted mean difference (WMD) of 0.35 servings/d (95% CI: 0.19, 0.52; *P* < 0.0001) increasing to a WMD of 0.59 servings/d (95% CI: 0.21, 0.98; *P* = 0.002) when contrasted against no education in 6 trials. Likewise for energy from total fat, 3 papers using TF compared with generic information showed a WMD of –2.2% (95% CI: –3.0, –1.4; *P* < 0.00001) and a WMD of –2.45% energy (95% CI: –4.08, –0.82; *P* = 0.0005) in the 6 cases where the control was presented with no information.

Michie et al. (9) classified behavioral techniques in healthy eating and PA. Uniting both of these interventions, they found an effect size of 0.41 (95% CI: 0.29, 0.52) in the 46 cases using SM compared with 0.26 (95% CI: 0.21, 0.30) in the 76 which did not use SM (*P* = 0.189). On the other hand, the effect size was 0.32 (95% CI: 0.24, 0.39) in the feedback domain (*n* = 61), as opposed to 0.30 (95% CI: 0.24, 0.37) in the other 61 occurrences. In a different analysis, healthy eating was looked at separately from PA, though SM was not examined independently but combined with the following: *1*) provision of feedback on performance; *2*) prompt intention formation; and *3*) specific goal-setting or review of behavioral goals. In these 13 trials, the effect size grew to 0.54 (95% CI: 0.21, 0.86), in contrast to 0.24 (95% CI: 0.18, 0.29) in the remaining 40 studies.

### Strengths

As mentioned above, compared with others, our review includes a broader range of population characteristics, outcomes, and intervention types despite focusing purely on the area of diet. The multilevel meta-analysis meant that we could include multiple dietary outcome results from single studies and could include results from different dietary outcomes across different studies. This greatly increased the statistical power of our meta-analysis and expanded the range of our systematic review, under the assumption that the different interventions were comparable examples of SM or TF (68). Due to the large amount of heterogeneity in the intervention designs, we conducted random-effects meta-analysis.

### Limitations

As we did not consult gray literature, we may have not identified all the relevant literature. Moreover, although the studies comprised a wide array of participants, environments, and modalities, they were only performed in high-income countries and mostly using nondigital methods, and therefore caution needs to be exercised in extrapolating the conclusions. Additionally, our results may be affected by misreporting; dietary intake is well known to be underreported (69) and this is not helped by having no consensus over the questionnaire or diary used for recording outcomes.

Finally, we were not able to assess long-term effectiveness of SM and TF as we only analyzed the data at the 6-mo stage and did not consider any follow-up results. Indeed, in comparison to other methods that are not SM or TF, it has been postulated that there is no sustained change long term, and when used for weight management, a decline in adherence tends to occur after 1 mo (56). Perhaps this is due to shifting motivations from those who prompted the initial change or an increased requirement of limited self-regulatory effort as automatic habitual responses are not yet fixed (65, 70).

Due to the sparse use of SM as an intervention on its own twice and together with TF in 3 trials, it is difficult to draw conclusions about its effectiveness, alone or combined. Additionally, we did not measure other intervention methods used in the trials such as goal setting (7), and as such it is hard to distinguish the individual contribution to the overall effect.

### Recommendations

We noticed that no studies had fiber as an outcome, despite evidence that an increased fiber intake can have health benefits (2). There were no trials in low- or middle-income countries. Therefore, we recommend the above areas for future research. It would also be beneficial to improve the reporting of group sizes, determine the reasons for drop-outs, ascertain the blinding procedures (particularly for data analysis) and consider how missing data have been dealt with so that discerning bias is easier. It will also be important to develop strategies to overcome the obstacles to using SM and TF, such as declining motivation, that have been identified.

### Conclusion

This systematic review of the literature set out to answer the question: are remote interventions that use SM or TF effective in changing eating behaviors? The meta-analysis showed a significant—albeit small, heterogeneous, and at risk of bias—positive effect of standalone SM and TF on dietary change (based predominantly on TF studies in this review). At a population level, such an intervention could have an appreciable impact. Given the ability of digital health interventions to deliver interventions remotely and to reach wide audiences (6), such interventions have the potential to make a contribution to improving the healthiness of diets.

## Supplementary Material

Supplemental data

## Notes

Supported by the NIHR Biomedical Research Centre, Oxford. PS is funded by a BHF Intermediate Basic Science Research Fellowship (FS/15/34/31656).

Supplemental Table 1 and Supplemental Figures 1 and 2 are available from the “Supplementary data” link in the online posting of the article and from the same link in the online table of contents at https://academic.oup.com/ajcn/.

**Abbreviations used:**
EDNP: Energy dense, nutrient poor
GP: General Practice
PA: physical activity
SBCT: self-regulatory behavior change techniques
SM: self-monitoring
SMD: standardized mean difference
SSB: sugar-sweetened beverage
TF: tailored feedback
WMD: weighted mean difference.
