# Supplementary material for: Systematic review and meta-analysis of remotely delivered interventions using self-monitoring or tailored feedback to change dietary behavior
Source: Am J Clin Nutr. 2018 Feb 26;107(2):247–56. doi: 10.1093/ajcn/nqx048 (PMC5875102; doi:10.1093/ajcn/nqx048)
Supplement: Supplemental data [file nqx048_supp.zip › ajcn163683-file001.docx]

**Systematic review and meta-analysis of remotely delivered interventions using self-monitoring or tailored feedback to change dietary behavior** Natalie Teasdale, Ahmed Elhussein, Frances Butcher, Carmen Piernas, Gill Cowburn, Jamie Hartmann-Boyce, Rhea Saksena, Peter Scarborough

**Supplemental Table 1:** Results of the multilevel univariate analyses exploring sources of heterogeneity

| ***Variable*** |  | ***Effect size*** | ***Standard error*** | ***p*** |
| --- | --- | --- | --- | --- |
| Delivery of intervention (baseline: mobile phone) | Website | -0.173 | 0.148 | 0.241 |
|  | Other | -0.164 | 0.143 | 0.251 |
| Risk of bias (baseline: higher risk of bias | Low risk of bias | -0.002 | 0.095 | 0.984 |
| Dietary outcome (baseline: fatty acid) | Fruit or veg | 0.009 | 0.036 | 0.813 |
|  | Other | 0.092 | 0.052 | 0.077 |
| Geography (baseline: Europe) | USA | -0.088 | 0.085 | 0.302 |
|  | Other | 0.146 | 0.113 | 0.195 |
| Length of intervention (continuous variable measured in months) | | -0.017 | 0.015 | 0.237 |
| Measurement method (baseline: food diaries) | FFQ | -0.141 | 0.090 | 0.115 |
|  | Other | 0.299 | 0.137 | 0.028 |
| Population type (baseline: general population) | Specified by risk factor | 0.144 | 0.112 | 0.198 |

*‘Effect size’ refers to the effect of each variable on the Standardised Mean Difference from the 50 dietary outcomes nested in 22 studies in a multilevel univariate analysis. Note that the outlying study (Mummah, 2016) was omitted from these analyses. Also note that each variable was included in a separate multilevel analysis with the Standardised Mean Difference as the outcome variable. FFQ = Food Frequency Questionnaire*
