# Supplementary material for: Systematic review and meta-analysis of remotely delivered interventions using self-monitoring or tailored feedback to change dietary behavior
Source: Am J Clin Nutr. 2018 Feb 26;107(2):247–56. doi: 10.1093/ajcn/nqx048 (PMC5875102; doi:10.1093/ajcn/nqx048)
Supplement: Supplemental data [file nqx048_supp.zip › ajcn163683-file002.docx]

**Systematic review and meta-analysis of remotely delivered interventions using self-monitoring or tailored feedback to change dietary behavior** Natalie Teasdale, Ahmed Elhussein, Frances Butcher, Carmen Piernas, Gill Cowburn, Jamie Hartmann-Boyce, Rhea Saksena, Peter Scarborough

**Supplemental Figure 1:** Forest plot of 15 dietary outcomes nested in 6 studies included in the meta-analysis restricted to studies at low risk of bias


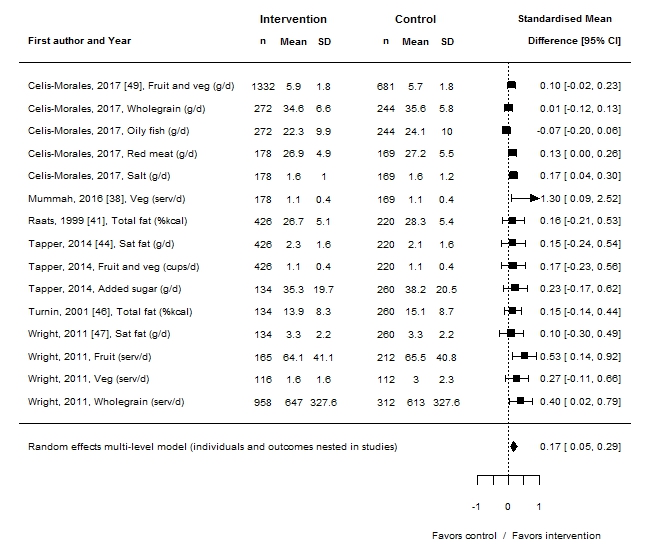


*g/d = grams per day; SD = standard deviation; serv/d = servings per day; %kcal = percentage of total*

I^2^ for heterogeneity = 0.432
