# Supplementary material for: Systematic review and meta-analysis of remotely delivered interventions using self-monitoring or tailored feedback to change dietary behavior
Source: Am J Clin Nutr. 2018 Feb 26;107(2):247–56. doi: 10.1093/ajcn/nqx048 (PMC5875102; doi:10.1093/ajcn/nqx048)
Supplement: Supplemental data [file nqx048_supp.zip › ajcn163683-file003.docx]

**Systematic review and meta-analysis of remotely delivered interventions using self-monitoring or tailored feedback to change dietary behavior** Natalie Teasdale, Ahmed Elhussein, Frances Butcher, Carmen Piernas, Gill Cowburn, Jamie Hartmann-Boyce, Rhea Saksena, Peter Scarborough

**Supplemental Figure 2:** Forest plot of 36 dietary outcomes nested in 17 studies included in the meta-analysis restricted to studies at higher risk of bias


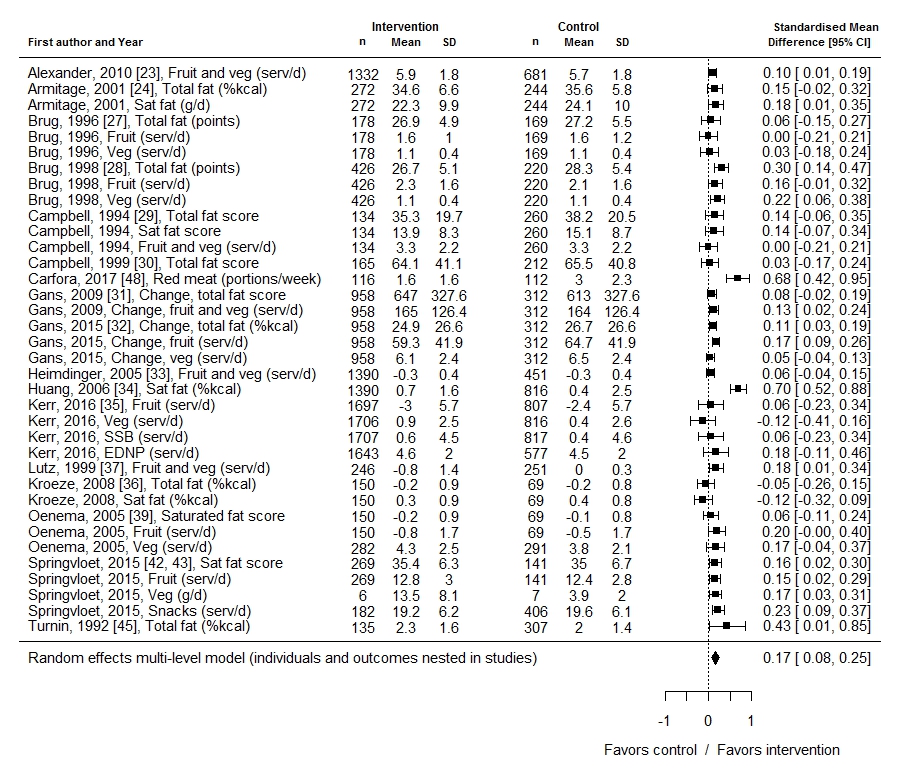


*EDNP = energy dense, nutrient poor; energy consumed; g/d = grams per day; SD = standard deviation; SSB = sugar sweetened beverages; serv/d = servings per day; serv/wk = servings per week; %kcal = percentage of total; Fat scores/points: Brug 98– “The fat score that ranges between 12 and 60 is the result of a short food frequency questionnaire in which the frequency of use and portion size of the 12 main fat sources in the Dutch diet are assessed.”; Campbell 1994: “ Dietary fat and saturated fat scores were obtained by multiplying frequency of consumption (calculated as servings per day) by portion data for each item and summing the items"; Campbell 1999: “Dietary fat scores were obtained by multiplying frequency of consumption adjusted to daily intake (3, 2, 1, 0.5, 0.14, 0.07 and 0) by fat content per serving of each item and summing items”; Gans 2009:”The FHQ fat summary score was calculated by taking the mean of all behavioral FHQ questions....response categories for the behavioral questions were: 0 =almost always, 1 = often, 2 = sometimes, 3 = rarely, and 4 = never.”; Oenema 2005: “Answers to the [FFQ] items were converted into a fat score ranging from 0 to 80, reflecting total saturated fat intake”; Springvloet 2015 “Saturated fat intake was measured with a [FFQ]...Based on this questionnaire, fat points were calculated...The total “fat score” was based on 35...food products [which]... fat points were assigned for each product group, ranging from zero...-5 ( ...summed up to create a total fat points measure.”*

I^2^ for heterogeneity = 0.834
